# Patient satisfaction and quality of life in breast reconstruction: assessment of outcomes of immediate, delayed, and nonreconstruction

**DOI:** 10.1186/s13104-020-05058-6

**Published:** 2020-04-19

**Authors:** Hianga Fayssa Fernandes Siqueira, Jorge Luiz de Almeida Teixeira, Reginaldo da Silva Lessa Filho, Evânia Curvelo Hora, Filipe Ferreira Brasileiro, Kênya de Souza Borges, Érika de Abreu Costa Brito, Marcela Sampaio Lima, Adriane Dórea Marques, Alex Rodrigues Moura, Tarcizo Costa Figueiredo Júnior, Thiago Costa de Oliveira, Ana Alice Santana Vasconcelos, Carlos Anselmo Lima

**Affiliations:** 1 Graduate Program in Health Sciences, Federal University of Sergipe, Aracaju, Brazil; 2 Brazil University Hospital, EBSERH/Federal University of Sergipe, Aracaju, Brazil; 3 Federal University of Vale Do São Francisco, Paulo Afonso, Brazil

**Keywords:** Breast cancer, Mastectomy, Breast reconstruction, Quality of life

## Abstract

**Objectives:**

This study was conducted aiming to assess the quality of life and satisfaction of women who had mastectomy treated with and without breast reconstruction.

**Results:**

A total of 81 women who had mastectomy were included, of whom 53 (65.4%) underwent breast reconstruction. Quality of life was not significantly better in the reconstruction group than the nonreconstruction group. Satisfaction with the surgically operated breast, whether reconstructed or not, was positively correlated with quality of life (p = 0.004). There was no significant difference in quality of life between women with immediate and late reconstruction. This study showed that the satisfaction of patients with the operated breast, reconstructed or not, is more important in quality of life than whether the breast was reconstructed or not. When we analyzed the quality of life of women who had mastectomy who were dissatisfied with their unreconstructed breasts, we observed that nonreconstruction had a negative impact on the quality of life.

## Introduction

Concerns about the quality of life (QOL) of women who had mastectomy have generated interest in providing not only cancer treatment but also better living and health conditions for this population. Therefore, the present study sought to evaluate whether breast reconstruction is a significant factor of QOL in this population.

Breast cancer is the leading cause of cancer death among women in Brazil and worldwide. For 2018–2019, approximately 59,700 new cases of breast cancer are expected in Brazil, with a risk estimate of 56.33 cases per 100,000 women [1]. Globally, the estimate was 2.1 million cases for the year 2018 [2]. For the Northeast region of Brazil, the estimate was 38.74/100 thousand. For the state of Sergipe, according to the cancer registry of the population base of the state, the estimate for 2018 was 71.88/100,000, and in Aracaju, 41.03 for every 100,000 women [3].

Despite the increased incidence of breast cancer worldwide, there have been advances in early detection with mammography, in addition to increasingly effective therapies for breast cancer [4]. The estimated 5-year survival rate of 70% for patients with breast cancer in Aracaju is noteworthy; although it is a city in a developing country, it follows global trends [5].

Mastectomy can be indicated depending on the breast cancer type, the stage each women had, to patients who are not candidates for breast-conserving therapy, and for prophylactic purposes [6]. Although mastectomy can cause sequelae and physical and psychological for women, generate dissatisfaction, and bring social and family implications [7].

## Main text

### Methods

This is a cross-sectional, analytical study in which the initial sample consisted of 132 women with breast cancer undergoing radical mastectomy and/or breast reconstruction. The women were assessed at least 1year after surgery. They had been operated at the University Hospital of the Federal University of Sergipe.

Women who did not have the perceptual-cognitive ability to answer the questionnaire, those who did not agree to participate in the study, those we were not able to contact, those who were under chemotherapy or radiotherapy, and those who had died were excluded, resulting in a final sample of 81 patients.

The cognitive evaluation was done through the Mini Mental State Examination (MMSE) [8]. The MMSE is an instrument that provides information on different cognitive parameters, containing questions grouped into seven different categories, and each one has the objective of evaluating a specific cognitive “function”: temporal orientation, spatial orientation, three-word registration, attention and calculation, three-word recall, language, and visual constructive ability. We used the version validated for the Brazilian population [9]. We adopted the cut-off point of 24 for patients with more than 9 years of education, while the cut-off was 17 those with a lower educational level [10].

After the MMSE, we applied the WHOQOL-*bref* questionnaire, which was intended to assess the QOL. The WHOQOL-*bref* was validated for the Brazilian population [11]. It is a self-assessed and self-explanatory instrument of 26 questions, 24 of which are classified into 4 domains: physical, psychological, social relationships, and the environment. The other two questions are general questions about the individual’s QOL and their satisfaction with their own health. Each question is scored in integers from one to five, and the lowest scores are assigned to the lowest/worst QOL. The mean of each domain was obtained at the end.

A question related to the degree of satisfaction with the reconstructed breast was added at the end of the questionnaire, and this degree was rated as very dissatisfied, dissatisfied, neither dissatisfied nor satisfied, satisfied, very satisfied. The concept of satisfaction oriented to the patients during the questionnaire was directed towards physical well-being in relation to the operated breast.

### Statistical analysis

The associations between categorical variables were evaluated using the Pearson Chi square test. Differences in the measures of central tendency were assessed using the Mann-Whitney and Kruskal-Wallis tests, in addition to the Dunn-Kruskal-Wallis test in multiple comparisons. The differences in the correlations were evaluated using analysis of covariance (ANCOVA). The significance level adopted was 5%, and the software used was the R Core Team 2019.

### Results

The present study comprised a final sample of 53 women with reconstructed breasts and 28 women with nonreconstructed breasts. These women had a mean age of 48.5 years (SD = 9.9 years), and the most frequent age group was 40 to 50 years (35.8%). Of all women interviewed, five had bilateral mastectomy, three were reconstructed, and two were not. A total of 45.7% of them finished high school, 50.6% were single, and 65.4% had an income of less than or equal to one Brazilian minimum wage (BM), which is equivalent to approximately 250 dollars. Among those surveyed, 45.0% considered their own health as “good,” and 54.3% reported not having comorbidities. When comparing the epidemiological characteristics of reconstructed and nonreconstructed patients, the groups were similar and there were no statistically expressive differences in the WHOQOL scores.

The average length of time of the application of the questionnaire after breast surgery was 3.8 years (SD = 3.2 years) for mastectomy and 2.5 years (SD = 1.1 years) for breast reconstruction.

Considering the women who were reconstructed, 47.2% (n = 25) were single, 67.9% (n = 36) were reconstruct immediately after mastectomy, at a mean age of 47.5 years (SD = 9.7), and 32.1% bilaterally, at a mean age of 51.4 years (SD = 10). Some 43.4% of reconstructions were done with subpectoral silicone implants, 35.8% with myocutaneous flaps of the latissimus dorsi muscle with breast implants, 11.3% with subpectoral tissue expanders, 5.7% with the transverse rectus abdominis flap (TRAM flap), and 3.8% with another type of reconstruction.

The average length of hospital stay among women who had mastectomy was 2.5 days (SD = 2.3 days), while the average length of hospital stay after breast reconstruction was 2.7 days (SD = 2.5 days). Among the 28 women who did not undergo breast reconstruction, 53.6% (15) were single, and 13 (46.4%) reported not wanting to reconstruct.

The QOL between the reconstructed patients and nonreconstructed patients were not significant, and the differences between patients with late and immediate reconstruction were also not statistically significant.

It is noteworthy that 77.4% (n= 41) of the women who had breast reconstruction did undergo radiation therapy, against 57% (n=16) of the women without breast reconstruction. The QOL measurements among patients who underwent neoadjuvant and / or adjuvant therapies with radiotherapy and chemotherapy, did not show statistically significant differences (Table 1).

**Table 1:** Clinical, socioeconomic, and treatment characteristics of patients related to the quality of life of 81 patients subjected to treatment for breast cancer

	Breast reconstruction		WHOQOL				
	Yes *n*	No *n*	Physical mean (SD)	Psychological mean (SD)	Social Relations mean (SD)	Environment mean (SD)	General mean (SD)
Age group, Mean (SD)	47.5 (9.7)	51.4 (10)					
	0.161 ^W^						
< 40	13	2	62.29 (11.02)	70.67 (11.9)	77.78 (13.25)	63.67 (13.56)	66.78 (9.68)
40–50	22	7	66.11 (13.24)	72.64 (9.73)	71.03 (16.06)	65.09 (8.36)	68.02 (8.88)
50-60	17	9	65.82 (10.49)	72.05 (9.38)	70.26 (18.59)	64..42 (11.19)	67.47 (9.59)
> 60	7	4	63.12 (9.77)	66.67 (14.68)	64.85 (14.01)	60.91 (12.11)	63.48 (9.16)
p-value	0.426^Q^		0.682 ^k^	0.679 ^k^	0.253 ^k^	0.660 ^k^	0.461 ^k^
Educational level							
Until elementary school	22	12	62.77 (10.72)	70.59 (11.59)	67.25 (17.7)	64.63 (12.75)	65.91 (9.83)
Until high school	29	8	65.25 (11.52)	70.81 (10.35)	72.97 (15.23)	62.91 (9.42)	66.82 (8.96)
Until graduation or superior	8	2	70.86 (12.62)	75.33 (9.58)	78 (13.35)	66.25 (8.19)	71.33 (7.6)
p-value	0.373^Q^		0.330 ^k^	0.399^k^	0.126^k^	0.464^k^	0.216^k^
Marital status							
Single	25	15	64.07 (11.89)	70..25 (11.99)	71 (16.64)	65.31 (11.95)	66.9 (10.34)
Not single	28	13	65.71 (11.12)	72.28 (9.5)	71.38 (16.28)	62.8 (9.46)	67.09 (8.15)
p-value	0.352^Q^		0.537^w^	0.582^w^	0.875^w^	0.234^w^	0.857^w^
How is your health?							
Weak	2	0	57.14 (8.08)^a.b^	65.00 (7.07)^a.b^	43.33 (14.41)^b^	51.25 (1.77)^a.b^	55.42 (2.95)^b^
Not bad, not good	16	9	58.17 (8.24)^b^	63.47 (10.43)^b^	64.27(17.41)^a,b^	58.80 (9.69)^b^	60.47 (7.99)^b^
Good	25	11	68.49 (9.43)^a^	75.19 (9.74)^a^	74.44 (11.76)^a^	67.36 (10.42)^a^	70.53 (7.88)^a^
Very good	15	2	68.07 (15.46)^a^	75.1 (7.56)^a^	76.08 (17.17)^a^	65.29 (9.76)^a^	69.90 (8.29)^a^
p-value	0.268^Q^		*0.002*^*k*^	*<0.001*^*k*^	*0.016*^*k*^	*0.002*^*k*^	*<0.001*^*k*^
Comorbidities							
Yes	23	14	64.48 (12.48)	70.00 (13.26)	68.65 (18.85)	63.78 (11.94)	66.15 (11.00)
No	36	8	65.26 (10.67)	72.35 (8.35)	73.33 (13.79)	64.26 (9.80)	67.71 (7.51)
p-value	0.048^Q^		0.661^w^	0.721^w^	0.181^w^	0.830^w^	0.605^w^
Family income							
Até 1 BM	40	13	63.67 (11.11)	69.94 (10.28)	70.44 (16.36)	62.5 (11.27)	65.69 (9.22)
De 1 a 2 BM	12	6	65.08 (8.69)	72.78 (11.90)	72.59 (17.84)	65.97 (10..71)	68.24 (9)
>2 BM	7	3	71.14 (16.13)	75.67 (10.89)	72.67 (14.89)	68.75 (5.8)	71.67 (8.8)
p-value	0.751^Q^		0.540 ^k^	0.131 ^k^	0.549 ^k^	0.093 ^k^	0.117 ^k^
Mastectomy technique							
Skin and ANC non-sparing	26	25	65.71 (10.84)	71.31 (11.51)	71.37 (17.35)	63.14 (10.12)	66.96 (9.12)
Skin sparing	15	3	65.4 (10.18)	71.67 (11.04)	73.7 (13.03)	66.11 (12.4)	68.24 (9.57)
Skin and ANC sparing	10	0	60.29 (15.68)	71.33 (6.32)	64 (16.09)	64 (9.66)	64.75 (8.53)
Other	2	0	62.86 (20.2)	66.67 (14.14)	80 (18.86)	68.75 (22.98)	67.92 (19.45)
p-value			0.509 ^k^	0.921 ^k^	0.471 ^k^	0.557 ^k^	0.755 ^k^
Breast reconstruction							
Yes			65.62 (11.74)	71.69 (9.97)	72.77 (15.82)	64.62 (10.88)	67.7 (9.02)
No			62.99 (10.71)	70.15 (12.91)	66.97 (17.39)	62.5 (10.55)	65.11 (9.76)
p-value			0.536^w^	0.616^w^	0.166^w^	0.343^w^	0.310^w^
Neoadjuvant therapy							
Chemotherapy	30	8	63.68 (12.12)	71.4 (11)	71.4 (16.18)	64.54 (11.36)	66.86 (9.22)
Chemotherapy and radiotherapy	2	1	81.9 (19.02)	71.11 (18.36)	71.11 (3.85)	61.67 (8.78)	71.11 (13.39)
None	27	13	64.79 (9.38)	71.17 (10.31)	71 (17.32)	63.75 (10.52)	66.81 (9.16)
p-value	0.509^Q^		0.230 ^k^	0.935 ^k^	0.991 ^k^	0.809 ^k^	0.704 ^k^
Adjuvant therapy							
Chemotherapy	9	2	62.6 (6.32)	71.52 (8.35)	70.3 (10.05)	64.77 (7.54)	66.52 (6.08)
Radiotherapy	24	6	64.29 (12.53)	71.44 (12.43)	72.44 (16.49)	64.08 (11.83)	67.03 (10.03)
Chemotherapy and radiotherapy	17	10	65.71 (12.45)	71.36 (11.26)	70.12 (19.16)	63.61 (12.45)	66.98 (10.69)
None	9	4	66.59 (10.84)	70.51 (8.26)	71.28 (15.49)	64.23 (7.1)	67.37 (6.88)
p-value	0.450^Q^		0.687 ^k^	0.957 ^k^	0.981 ^k^	0.894 ^k^	0.993 ^k^
Reconstruction technique							
TRAM flap	3		74.29 (17.38)	76.67 (6.67)	62.22 (25.24)	65 (10.9)	70.28 (11.82)
Myocutaneous flaps of the Latissimus Dorsi Muscle with breast implants	19		66.57 (8.7)	73 (10.7)	79 (16.08)	66.5 (11.65)	69.71 (8.29)
Subpectoral silicone implants	23		63.81 (12.83)	70 (9.56)	69.14 (12.56)	63.61 (9.94)	65.96 (8.4)
Subpectoral tissue expanders	6		66.94 (12.33)	73.81 (10.44)	71.43 (19.52)	61.79 (11.25)	67.5 (11.1)
Another technique	2		62.86 (20.2)	66.67 (14.14)	80 (18.86)	68.75 (22.98)	67.92 (19.45)
p-value			0.646 ^k^	0.721 ^k^	0.203 ^k^	0.751 ^k^	0.635 ^k^
Momento of breast reconstruction							
Immediate	36		63.83 (12.35)	70.24 (10.31)	73.01 (15.2)	64.82 (11.34)	66.91 (9.67)
Delayed	17		69.68 (9.3)	75 (8.5)	72.22 (17.6)	64.17 (10.04)	69.49 (7.26)
Nonreconstruction	28		62.99 (10.71)	70.15 (12.91)	66.97 (17.39)	62.5 (10.55)	65.11 (9.76)
p-value			0.084 ^k^	0.228 ^k^	0.382 ^k^	0.611 ^k^	0.327 ^k^
Complications							
Mastectomy		6	54.76 (9.97)^a^	65.71 (12.87)	61.9 (23.32)	59.29 (9.97)	59.76 (10.84)
Immediate reconstruction	17		64.87 (13.54)^a.b^	69.17 (5.56)	65.83 (9.72)	60.94 (7.06)	64.69 (6.41)
Delayed reconstruction	7		73.88 (9.82)^b^	73.33 (8.82)	68.89 (7.7)	55.83 (10.1)	63.06 (9.18)
None	29	22	64.87 (10.32)^a.b^	73.06 (10.58)	75.56 (19.56)	66.88 (11.97)	71.18 (10.14)
p-value			*0.017* ^k^	0.248 ^k^	0.539 ^k^	0.792 ^k^	0.359 ^k^

The variables in italics had statistically significant values (p <0.05)

*SD* standard deviation, *N* absolute frequency, *TRAM flap* transverse rectus abdominis flap, *BM* Brazilian minimum wage

^w^Mann-Whitney test; ^K^Kruskal-Wallis test; ^Q^Pearson’s Chi square test

The longer hospitalization time after mastectomy had a significant negative correlation with the scores WHOQOL-*bref* in the physical, psychological, and general domains (ρ = −0.259; p = 0.020). Longer hospitalization time after breast reconstruction had a negative impact in the psychological domain (ρ = −0.301; p = 0.023) and the number of radiation therapy sessions had no impact on scores of physical, psychological, social relations and general (Table 2).

**Table 2:** Correlations between duration of surgical treatment, number of adjuvant radiotherapy sessions, length of hospital stay after surgery, and perception of quality of life and health and the WHOQOL-*bref* questionnaire scores

	WHOQOL				
	Physical mean (SD)	Psychological mean (SD)	Social relations mean (SD)	Environment mean (SD)	General mean (SD)
Time after mastectomy	0.063 (0.582)	−0.006 (0.96)	−0.071 (0.529)	−0.107 (0.344)	−0.031 (0.783)
Time after reconstruction	−0.066 (0.619)	−0.093 (0.479)	−0.133 (0.312)	−0.071 (0.589)	−0.094 (0.473)
Adjuvant radiotherapy sessions	0.076 (0.498)	0.078 (0.490)	0.035 (0.756)	−0.085 (0.451)	0.046 (0.684)
Hospitalization time after mastectomy	*−0.293 (0.008)*	*−0.325 (0.003)*	−0.092 (0.414)	−0.075 (0.508)	*−0.259 (0.020)*
Hospitalization time after reconstruction	−0.141 (0.295)	*−0.301 (0.023)*	0.028 (0.838)	0.074 (0.585)	−0.101 (0.456)
Perception of quality of life	*0.492 (<0.001)*	*0.477 (<0.001)*	*0.357 (0.001)*	*0.489 (<0.001)*	*0.571 (<0.001)*
Perception of health	*0.404 (<0.001)*	*0.54 (<0.001)*	*0.256 (0.021)*	*0.502 (<0.001)*	*0.542 (<0.001)*

*P* Spearman correlation. The variables in italic had statistically significant values (p <0.05)

Regarding satisfaction with the operated breast, Table 3 shows that the desire of the woman to undergo breast reconstruction but for some reason being prevented from undergoing it was associated with a lower score on the physical domain (ρ = −0.654; p = 0.029), the social relationship domain (ρ = −0.643; p = 0.033), and the overall WHOQOL-*bref* (ρ = −0.673; p = 0.023).

**Table 3:** Satisfaction regarding the operated breast between reconstructed and nonreconstructed patients and the correlation with the WHOQOL-*bref*

	WHOQOL				
	Physical mean (SD)	Psychological mean (SD)	Social Relations mean (SD)	Environment mean (SD)	General mean (SD)
Satisfaction of patients with their operated breasts					
Total	0.189 (0.090)	*0.375 (0.001)*	*0.286 (0.010)*	*0.289 (0.009)*	*0.317 (0.004)*
With breast reconstruction	0.231 (0.097)	*0.419 (0.002)*	0.185 (0.184)	0.181 (0.195)	*0.300 (0.029*)
Nonreconstructed but desired it	*−0.654 (0.029)*	−0.462 (0.153)	*−0.643 (0.033)*	−0.071 (0.835)	*−0.673 (0.023)*
Non reconstructed and don’t desired it.	0.373 (0.210)	0.486 (0.093)	0.504 (0.079)	0.297 (0.324)	0.487 (0.091)
ANCOVA	0.054	*0.036*	*0.025*	0.500	*0.023*

*P* Spearman correlation, *ANCOVA* Analysis of covariance. The variables in italic had statistically significant values (p <0.05)

Within the group of reconstructed patients, we found that the patients who had greater satisfaction with their breasts had higher the psychological domain score (ρ = 0.419; p = 0.002) and higher overall QOL score (ρ = 0.300; p = 0.029).

The satisfaction of patients with their operated breasts, whether reconstructed or not reconstructed, had a positive impact in the psychological domain (ρ = 0.375; p = 0.001), in social relationships (ρ = 0.286; p = 0.010), in environment (ρ = 0.289; p = 0.009) and the overall WHOQOL-*bref* (ρ = 0.317; p = 0.004) (Table 3).

### Discussion

When we analyzed the QOL of women who underwent mastectomy with breast reconstruction, no significant difference was observed compared to women who had mastectomy without breast reconstruction. This result corroborates the data of a meta-analysis published in 2009, all high-quality studies in that meta-analysis found QOL, body image, or sexual image equivalent or worse in women who underwent mastectomy with reconstruction compared with women who underwent mastectomy only [12]. However, when we analyzed the QOL of nonreconstructed women who wished to reconstruct their breasts, we found that nonreconstruction showed a significant negative correlation with general QOL, as well as in the physical and social relationships domains. Based on these data, we believe that although the group of women who undergo breast reconstruction do not have significant differences in the QOL compared to nonreconstructed women, nonreconstruction can negatively impact the QOL when these women express the desire to reconstruct.

The satisfaction with the operated breast of the reconstructed patients also had a positive correlation with general QOL, with a higher correlation in the psychological domain. Matthews et al. (2017) showed that women with greater psychological well-being were more likely to report greater satisfaction with the appearance of the breast, and satisfaction with the appearance of the breast promoted greater psychosocial well-being [14]. The results of this study and those of Matthews et al. [13] indicate that satisfaction with the reconstructed breast is an important factor for a better QOL, especially when considering the psychological aspect; and although the other domains, physical, social relations, and environment, did not have significant correlations, the direction of the effects on them was positive.

In the present study, satisfaction with the operated breast was correlated with QOL whether the patient underwent breast reconstruction or not. These data may be linked to personal feelings for or against reconstruction or individual motivations, such as the desire to regain their femininity, improve their body image, or avoid additional surgery [14–16].

Comparing the QOL of women with immediate vs. late reconstruction, we did not find significant differences in QOL scores, but we found that the mean of the physical domain was the mean that had the greatest difference between these women. In contrast, Zhong et al. (2016) showed that mastectomy with immediate breast reconstruction can protect breast cancer patients from a period of psychosocial suffering, dissatisfaction with body image, and dissatisfaction with sexual life compared with those who underwent late reconstruction [17].

Dauplat et al. (2017), in a multicenter study, using another instrument for analysis of QOL, found that mastectomy followed by reconstruction preserved the QOL, but only if reconstruction was proposed for certain types of patients, such as young age, among others [18]. In our study, most of the reconstructed women underwent immediate reconstruction, they were over 40 years, so age could have been a negative impact factor. However, women with late reconstruction, whose mean age was greater than 50 years, reported better scores in the physical, psychological, and general domains.

Patients with longer hospitalization time after mastectomy had lower WHOQOL-*bref* scores in the physical, psychological, and general domains. Longer hospitalization time after breast reconstruction had a negative impact only on the psychological domain. These data are interesting because although the event occurred in the past, it still has an impact on the QOL of these women, especially in the psychological setting.

Our results may converge with those of Colakoglu et al. they found that women who had complications had lower aesthetic satisfaction compared to patients who did not. When analyzed by the time of complication onset, patients with early complications had significantly lower aesthetic satisfaction scores than patients without complications [19].

### Conclusions

The present study showed that the satisfaction of patients with the operated breast, reconstructed or not, is more important in QOL than whether the breast was reconstructed or not. When we analyzed the QOL of women who were dissatisfied with their unreconstructed breasts, we observed that nonreconstruction had a negative impact on the QOL.

## Limitations

The present research was a cross-sectional study and therefore we cannot test the impact of breast reconstruction on quality of life, this could be clarified in a prospective study. The sexual orientation of patients has not been evaluated, and this may have interfered with the results, as the literature shows that women who identify themselves as lesbians or gays are generally not interested in reconstruction [20].

## Data Availability

The datasets used and/or analyzed during the current study are available from the corresponding author on reasonable request.

## Abbreviations

ANCOVA: Analysis of covariance
K: Kruskal Wallis test
MMSE: Mini mental state examination
N: Absolute frequency
P: Spearman correlation
Q: Pearson’s Chi square test
QOL: Quality of life
SD: Standard deviation
TRAM flap: Transverse rectus abdominal flap
W: Mann Whitney test
